# Towards a genome-scale kinetic model of cellular metabolism

**DOI:** 10.1186/1752-0509-4-6

**Published:** 2010-01-28

**Authors:** Kieran Smallbone, Evangelos Simeonidis, Neil Swainston, Pedro Mendes

**Affiliations:** 1 Manchester Centre for Integrative Systems Biology, Manchester Interdisciplinary Biocentre, 131 Princess Street, Manchester, M1 7DN, UK; 2 School of Mathematics, The University of Manchester, Oxford Road, Manchester M13 9PL, UK; 3 School of Chemical Engineering and Analytical Science, The University of Manchester, Oxford Road, Manchester M13 9PL, UK; 4 School of Computer Science, The University of Manchester, Oxford Road, Manchester M13 9PL, UK; 5 Virginia Bioinformatics Institute, Virginia Tech, Washington Street 0499, Virginia 24061, USA

## Abstract

**Background:**

Advances in bioinformatic techniques and analyses have led to the availability of genome-scale metabolic reconstructions. The size and complexity of such networks often means that their potential behaviour can only be analysed with constraint-based methods. Whilst requiring minimal experimental data, such methods are unable to give insight into cellular substrate concentrations. Instead, the long-term goal of systems biology is to use kinetic modelling to characterize fully the mechanics of each enzymatic reaction, and to combine such knowledge to predict system behaviour.

**Results:**

We describe a method for building a parameterized genome-scale kinetic model of a metabolic network. Simplified linlog kinetics are used and the parameters are extracted from a kinetic model repository. We demonstrate our methodology by applying it to yeast metabolism. The resultant model has 956 metabolic reactions involving 820 metabolites, and, whilst approximative, has considerably broader remit than any existing models of its type. Control analysis is used to identify key steps within the system.

**Conclusions:**

Our modelling framework may be considered a stepping-stone toward the long-term goal of a fully-parameterized model of yeast metabolism. The model is available in SBML format from the BioModels database (BioModels ID: MODEL1001200000) and at http://www.mcisb.org/resources/genomescale/.

## Background

Recent advances in genome sequencing techniques and bioinformatic analyses have led to an explosion of systems-wide biological data. In turn, the reconstruction of genome-scale networks for micro-organisms has become possible. Whilst the first stoichiometric models were limited to the central metabolic pathways, later efforts such as iFF708 [1] and iND750 [2] were much more comprehensive. A recent community-driven reaction network for *S. cerevisiae *(bakers' yeast) consists of 1761 reactions and 1168 metabolites [3].

The ability to analyse, interpret and ultimately predict cellular behaviour is a long sought-after goal. The genome sequencing projects are defining the molecular components within the cell, but describing their integrated function will be a challenging task. Ideally, one would like to use enzyme kinetics to characterize fully the mechanics of each reaction, in terms of how changes in metabolite concentrations affect local reaction rates. However, a considerable amount of data and effort is required to parameterize even a small mechanistic model; the determination of such parameters is costly and time-consuming, and moreover much of the required information may be difficult or impossible to determine experimentally. Instead, genome-scale metabolic modelling has relied on constraint-based analysis [4], which uses physicochemical constraints such as mass balance, energy balance, thermodynamics and flux limitations to describe the potential behaviour of an organism. Such methods, however, ignore much of the dynamic nature of the system and are unable to give insight into cellular substrate concentrations. These methods are more suitable for defining the wider limits of systems behaviour than making reliable and accurate predictions about metabolism.

In a previous paper, we presented a method for constructing a kinetic model for a metabolic pathway based only on the knowledge of its stoichiometry [5]. Here, we present a first attempt at the creation of a parameterized, genome-scale kinetic model of metabolic networks, through appending existing kinetic models of constituent metabolic pathways from the BioModels database [6] to a stoichiometric model of yeast metabolism [3]. The results (see Additional file 1) are presented in SBML (Systems Biology Markup Language; http://sbml.org/) [7], using MIRIAM-compliant annotations (Minimal Information Requested In the Annotation of Models; http://www.ebi.ac.uk/miriam/) [8]. Critically, such markup allows automated reasoning about the model's assumptions and provenance.

## Results and Discussion

### Algorithm

#### Model construction

A number of reconstructions of the metabolic network of yeast based on genomic and literature data have been published. However, due to different approaches utilized in the reconstruction, as well as different interpretations of the literature, the earlier reconstructions differ significantly. A community effort resulted in a consensus network model of yeast metabolism, combining results from previous models ([3], available from http://www.comp-sys-bio.org/yeastnet). In all, the resulting consensus network consists of 1857 reactions (of which 1761 are metabolic) involving 2153 chemical species (of which 1168 are metabolites). Species in the model are annotated using both database-dependent (*e.g*. ChEBI [9]) and database-independent (*e.g*. InChI [10]) references, generating for the first time a representation that allows computational comparisons to be performed.

Species are localized to 15 compartments, including membranes. To limit complexity, we decompartmentalize the model, restricting entities to intra- or extra-cellular space. We also lump together reactions catalyzed by isoenzymes; the resultant model is reduced in size to 1059 reactions, of which 956 are metabolic, involving 1748 species, of which 820 are metabolites (the remaining 938 species are enzymes and enzyme complexes). Estimation of unknown system fluxes are addressed with the use of flux balance analysis (FBA) [11]. FBA allows the identification of an optimal path through the network in order to achieve a particular objective, assuming (in a biological sense) that the organism has evolved towards maximal metabolic efficiency, within its underlying physicochemical, topological, environmental and regulatory constraints [12]. Mathematically, FBA is framed as a linear programming (LP) problem(1)

That is, we define an objective function *Z*, a linear combination of the fluxes *v*_*j*_, that we maximize over all possible steady state fluxes (*N v *= 0; where *N *is the *m × n *stoichiometric matrix) satisfying certain constraints. In many genome scale metabolic models, a biomass production reaction is defined explicitly that may be taken as a natural form for the objective function. The metabolic reconstruction used here [3] lacks such a sink for metabolism. We accomplish this by adding a pseudo-reaction representing cellular growth (sometimes referred to as "biomass production"). The biomass composition used here is taken from the iND750 model [2].

In a previous paper [5], we defined a method for the generation of kinetic models of cellular metabolism, based solely on the knowledge of reaction stoichiometries. This modelling framework requires little experimental data regarding variables and no knowledge of the underlying mechanisms for each enzyme; nonetheless it allows inference of the dynamics of cellular metabolite concentrations. The fluxes found through FBA are allowed to vary dynamically [13]. To create a kinetic model (of minimal complexity), four sets of information are required:

• Network stoichiometry (*N*).

• Reference fluxes (v*) through the network.

• Reference metabolite concentrations (x*).

• Elasticities (*ε*) -- changes in reaction rates with effector levels.

**Figure 1:**
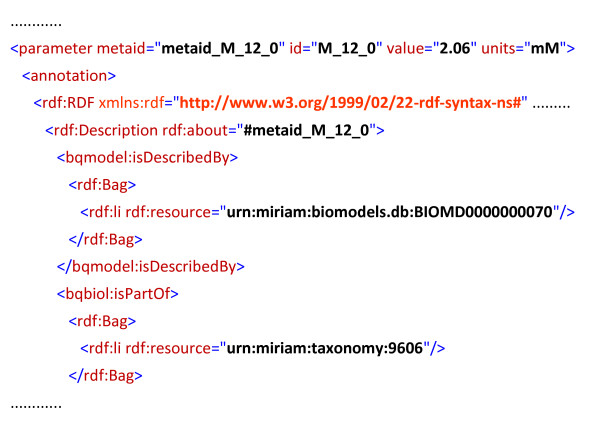
**An example of the SBML model's MIRIAM-compliant annotations**. The (concentration) parameter is taken from BioModels ID 70. Since the parameter is not available from yeast, it is flagged as originating from taxonomy 9606 (*H. Sapiens*).

To the stoichiometric model, we append kinetics (fluxes, concentrations and elasticities) from the set of models available from the BioModels database (11^th ^release) [6]. As metabolites in BioModels are annotated using computer-readable references, it was possible to curate the mapping to our stoichiometric model in a semi-automated manner. Where available, the parameters are taken as the median value from all yeast models. Where unavailable, these are taken from species other than yeast, or estimated as described below if not available for any species. An example of the SBML code used to mark up a typical kinetic parameter is presented in Figure 1.

#### Flux estimation

55 reactions in the (decompartmentalized) genome-scale model have fluxes that are defined in models stored on the BioModels database. Of these, the 21 data specific to yeast are presented in Table 1. For the rest of the flux space, our reference flux (v*) is found by solving the linear programming problem described in formulation 2 below, by minimizing the distance to these 55 target fluxes (*v*^*T*^):(2)

**Table 1:** Selected reaction fluxes used in the model

Reaction	Flux (mM/s)
acetaldehyde transport	0.00141
adenylate kinase	0
alcohol dehydrogenase, reverse rxn (acetaldehyde → ethanol)	1.17
ATPase, cytosolic	0.595
enolase	1.76
ethanol transport	0.0134
fructose-bisphosphate aldolase	0.733
glycerol-3-phosphate dehydrogenase (NAD)	0.149
glycerol-3-phosphatase	0.051
glyceraldehyde-3-phosphate dehydrogenase	1.06
glucose transport (uniport)	0.59
glycerol transport via channel	0.00141
hexokinase (D-glucose:ATP)	0.866
phosphofructokinase	0.606
glucose-6-phosphate isomerase	0.733
phosphoglycerate kinase	0.875
phosphoglycerate mutase	1.76
pyruvate kinase	1.06
pyruvate decarboxylase	1.25
triose-phosphate isomerase	0.395
alpha, alpha-trehalose-phosphate synthase (UDP-forming)	0.04

The data are taken from those models in the BioModels database specific to yeast.

where *BM *denotes the subset of *j *that includes all the reactions with fluxes defined in BioModels. A unique reference flux (see additional file 2) is chosen from the space of all solutions to the above problem, by finding the box that defines the maximum and minimum values attainable by each *v*_*j*_, then choosing a flux as close as possible to the centre of the box. Iterating, the method minimizes and centres the flux through the network and, in this case, fixes all 956 fluxes to unique values. The algorithm [14] that produces the unique solution from the available flux space is described briefly below.

A simple FBA formulation is solved, in order to identify the maximum achievable growth rate, Z*. For the first iteration, we minimize the total flux required to achieve Z*. This assumption (i.e. that the cell minimizes its total flux. [15]) may be posed as a LP problem by decomposing fluxes *v*_*j *_into their positive and negative parts. The solution of this first iteration provides the minimal total flux through the network (*Z*_1_). We then find the bounds on each reaction flux, subject to the new constraint that the total flux through the network cannot be larger than *Z*_1_. The bounds are calculated by solving an optimisation problem for maximizing and minimizing the flux of each reaction iteratively. These limits are set as the new upper and lower bounds for the fluxes. The "centre" for each flux is the mean of the new bounds, as the most representative value of all solutions.

In the second iteration, we place a box around the hull (defining new bounds), before minimizing the distance between the flux of each reaction and the centre value, subject to the constraint that the total network flux cannot exceed *Z*_1_, as found in the first iteration. In turn, this leads to new bounds and a corresponding centre. Each iteration of the algorithm adds an additional constraint, and the flux is drawn towards the centre of the bounds. After a finite number of iterations, the bounds converge to a single solution, within a specified tolerance.

The algorithm is explained in detail in a previous paper [14], which described a method for finding a unique solution within the space of all possible flux distributions in FBA. In that paper, the algorithm is used on four recent genome-scale metabolic reconstructions. Using an iteration of linear programs, unique flux solutions are found in the available flux space for each organism.

#### Concentrations

82 intracellular metabolites' concentrations are defined in various models within BioModels. Of these, the 22 specific to yeast are presented in Table 2. As concentrations must be given for all intracellular metabolites, the undefined remainder are set to the median concentration of ~0.18 mM. Extracellular metabolites are defined as in the "metabolic footprinting" medium [16] and reproduced in Table 3 for completeness.

**Table 2:** Selected intracellular metabolite concentrations used in the model

Metabolite	Concentration (mM)
3-Phospho-D-glyceroyl phosphate	2.75 × 10^-4^
D-Glycerate 2-phosphate	0.0371
3-Phospho-D-glycerate	0.278
Acetaldehyde	0.17
ADP	1.63
AMP	0.796
ATP	1.13
CO2	1
Dihydroxyacetone phosphate	0.59
Ethanol	50
D-Fructose 2,6-bisphosphate	0.02
D-Fructose 6-phosphate	0.112
D-Fructose 1,6-bisphosphate	2.82
Glyceraldehyde 3-phosphate	0.069
D-Glucose 6-phosphate	1.02
D-Glucose	0.0906
Glycerol	2.27
Glycerol 3-phosphate	0.457
Nicotinamide adenine dinucleotide	1.5
Nicotinamide adenine dinucleotide - reduced	0.0861
Phosphoenolpyruvate	0.0302
Pyruvate	8.36

The data are taken from those models in the BioModels database specific to yeast.

**Table 3:** Extracellular metabolite concentrations used in the model

Metabolite	Concentration (mM)
4-Aminobenzoate	0.0015
L-Arginine	1
L-Aspartate	1
Biotin	8.2 × 10^-5^
Citrate	1
Fumarate	1
D-Glucose	11.1
L-Glutamate	1
L-Histidine	1
myo-Inositol	0.055
potassium	7.11
L-Leucine	1
L-Lysine	1
L-Malate	1
L-Methionine	1
Sodium	1.71
Ammonium	38
(R)-Pantothenate	0.0042
Pyridoxine	0.0019
Pyruvate	1
Riboflavin	5.3 × 10^-4^
L-Serine	1
Sulfate	42.2
Succinate	1
Thiamin	0.0012
L-Threonine	1
L-Tryptophan	1
L-Valine	1

Values are as defined in the "metabolic footprinting" medium [16].

#### Elasticities

151 elasticities are calculated from models within BioModels, using symbolic differentiation. For the remaining values, we follow the tendency modelling approach of Visser *et al*. [17], whereby the elasticities are estimated as the negative of the corresponding stoichiometric coefficients [5]. The exception is irreversible reactions - products here are assumed to have no effect on reaction rates. These elasticities are identical to those that would be found through the assumption of mass action kinetics. Consider, as an example, an irreversible reaction

An assumption of irreversible mass-action kinetics would lead to reaction rate *v *= *k A*^2 ^*B *and hence elasticity , the negative of its stoichiometry (-2).

#### Linlog kinetics

To produce our genome-scale, kinetic model of yeast metabolism, the above parameters may be combined in a phenomenological rate law such as linlog kinetics:(3)

where *c *denotes the compartment volumes. The benefit of this approximation lies in the existence of analytic forms for steady states and their stability matrix [5], thus avoiding computational problems associated with models of this size [18]. In a recent investigation, the linlog approximation was proved better than its alternatives (linear, power laws, generic and convenience) at describing *E. coli *sugar metabolism [19].

### Testing

#### Control analysis

To test the resultant genome-scale model, and to try and indentify key steps in the metabolic network of yeast, we calculate the flux control coefficients for reactions, as defined by metabolic control analysis (MCA). MCA studies how the control of fluxes and intermediate concentrations in a metabolic pathway is distributed among the different enzymes that constitute the pathway. Developed independently by Kacser and Burns [20] and Heinrich and Rapoport [21], the main theorems of MCA were given rigorous theoretical backing by Reder [22]. Of particular interest is the connectivity theorem, highlighting the close relationship between the local properties of individual reactions (elasticities) and global properties of the system (control coefficients). This theorem links the properties of the individual reactions (elasticities) to the properties of the system (control coefficients).

Whilst Reder's formula is often used in computational applications, it assumes that a certain matrix is invertible; this may not be true, especially if some reference reaction rates are zero. For example, the number of independent metabolites is often defined solely in terms of stoichiometry as rank(*N*) (here = 616). However, once kinetics are taken into account, this number drops drastically to rank(*N*·diag(v*)·ε) = 205. Reder's method only holds if these two values are identical. Thus, in Methods, we derive again the main results of MCA without relying on such an assumption.

In Tables 4 and 5 we present those fluxes which have most control over glucose uptake and biomass production (which may be assumed proportional to growth), respectively (see additional files 3 and 4 for complete lists). The tables demonstrate the utility of the connectivity theorem, allowing calculation of global control coefficients from local elasticities. The results also demonstrate the necessity of genome-scale modelling when intimating system behaviour. For example, studying Table 4 (control over glucose transport), whilst one expects glycolytic reactions to exert strong control over glucose uptake, the regulation by L-asparaginase comes as a surprise. Also, from Tables 4 and 5, one can observe glucosamine-6-phosphate deaminase, glutamine-fructose-6-phosphate transaminase and glutamine synthetase at or near the top of both tables. These 3 reactions are closely related to the production of glutamate in amino acid metabolism. The *negative *control over glucose transport and the *positive *control over biomass production from these reactions would seem to suggest that an increase in their flux would increase growth while reducing glucose consumption. This is an example of the kind of hypotheses that can only be made with a genome-scale model, like the one produced using the methodology presented here. Such hypotheses can then be tested experimentally to help us expand our understanding of metabolism.

**Table 4:** Reactions exerting most control over glucose transport

Reaction	*C*^*J*^
glucose transport (uniport)	1.149
glucosamine-6-phosphate deaminase	-0.787
glutamine-fructose-6-phosphate transaminase	-0.655
glutamine synthetase	-0.520
inorganic diphosphatase	0.421
L-asparaginase	0.323
ATPase, cytosolic	0.250
phosphofructokinase	0.235
glycerol-3-phosphate dehydrogenase (NAD)	-0.233
adenylate kinase (GTP)	0.231

Reactions are ranked in terms of their flux control coefficient. See additional file 3 for the complete list.

**Table 5:** Reactions exerting most control over biomass production

Reaction	*C*^*J*^
glucosamine-6-phosphate deaminase	0.532
glutamine-fructose-6-phosphate transaminase	0.441
glutamine synthetase	0.358
H2O transport via diffusion	0.212
inorganic diphosphatase	-0.193
glycerol-3-phosphate dehydrogenase (NAD)	0.189
L-asparaginase	-0.146
adenylate kinase (GTP)	-0.142
glucose transport (uniport)	-0.132
ribonucleoside-triphosphate reductase (UTP)	-0.104

Reactions are ranked in terms of their flux control coefficient. See additional file 4 for the complete list.

### Implementation

The systems biology approach often involves the development of mechanistic models, such as the reconstruction of dynamic systems from the quantitative properties of their elementary building blocks. Typically, this is performed in a 'bottom-up' manner, whereby models built as individual elements are experimentally-determined. Here we propose an alternative, 'top-down' mechanism, whereby an approximative model of the whole system is built initially; this model can then be used to guide experimental design and can subsequently be updated as specific knowledge becomes available from experimental results, following the iterative 'cycle of knowledge' approach [23]. At any point of this iterative approach, detailed kinetic rate laws can be included if they become available, in which case the approach is then a hybrid top-down and bottom-up approach.

The genome-scale model that is produced with the presented methodology is offered in SBML format, with MIRIAM-compliant annotations. Such markup allows automated reasoning about the model's assumptions and provenance [24]. A variety of software programs (*e.g*. COPASI [25]) have been designed to interface with SBML, but do not generally encounter models of this size. Indeed, the kinetic model produced here has over an order of magnitude more metabolites and reactions than any other kinetic model found in the BioModels repository. As the field develops, so larger models will be built, and software programs will be required to interface with models of at least this size. Thus, this methodology also allows software testing and advancement. The presence of analytic solutions facilitates validation of new tools, and avoids the usual problems with the high demands on computational power that models of this size have.

## Conclusions

In this paper, we present a novel methodology that can be used to create a parameterized, genome-scale kinetic model of the metabolic network of an organism. The methodology is demonstrated by its application on yeast metabolism, through appending existing kinetic submodels from the BioModels database to a stoichiometric model of yeast. The final model has 956 metabolic reactions involving 820 metabolites and, to our knowledge has significantly wider scope than any previous models of comparable type. We demonstrate the usefulness of such a model, by applying the principles of metabolic control analysis to identify key steps within the network.

Critically, both the original stoichiometric model, and the kinetic model that constitutes the end-result of the method are available in SBML, using MIRIAM-compliant annotations. Models in BioModels are annotated with computer-readable references such as ChEBI [9] or InChI [10], which made it possible to curate the mapping to the stoichiometric model in a semi-automated manner. While fully-automated mapping of BioModels reactions to those in our stoichiometric model would be preferable, inconsistencies such as unbalanced reactions in either data resource prevent this at the current time. As systems biology is still a new and emerging field, it should be expected that discrepancies and other annotation issues will improve considerably. This, combined with greater availability of kinetic models for reactions and pathways in model repositories such as BioModels in the future, would mean that our methodology could be used to provide an increasingly more accurate and detailed genome-scale, kinetic model for an organism, in an efficient and automated manner. Furthermore, the approach should benefit from expanding its scope in order to exploit other resources containing kinetic data, such as SABIO-RK [26] and BRENDA [27].

Our methodology clearly has limitations, in that the linlog framework is only valid in a region near the chosen reference state. Moreover, due to the vast lack of information, many of the parameters used in building the model are unknown and must be estimated through techniques such as flux balance analysis. Nonetheless, our modelling framework is a necessary stepping stone at creation of a genome-scale kinetic model, and may thus be considered the first step in the deductive-inductive 'cycle of knowledge' crucial for systems biology [23]. We have demonstrated that this first model can be used to pinpoint, through sensitivity analysis, reactions that have the most control over the network, or reactions for which small perturbations of the values of their kinetic parameters lead to significant changes in the predictions of the model. Subsequent experimental work, such as kinetic assays may be used to improve the model's resolution. In the present case this includes glucosamine-6-phosphate deaminase, glutamine-fructose-6-phosphate transaminase and glutamine synthetase. The model (see additional file 1) is publically available for download in SBML format from the BioModels database (BioModels ID: MODEL1001200000) and at http://www.mcisb.org/resources/genomescale/.

## Methods

### Control analysis

Let us return to Equation (3), a generalized description of the temporal evolution of a metabolic network in differential equation format. Let us also assume that the reference state *x *= x* corresponds to a steady state - i.e. N v* = 0, where v* = *v*(x*). Writing  (for algebraic simplicity), *N *= diag(*c*)^-1^·*N *and , and dropping hats for convenience, we transform the system into the more recognisable form(5)

where *x *= 0 now corresponds to the steady state. Linearizing about this steady state(6)

where *ε*' is the *n × m *unscaled elasticity matrix.

In general, the rank(*N ε*') = *m*_0 _<*m *and the system defined above will display moiety conservations - certain metabolites can be expressed as linear combinations of other metabolites in the system. Note that the number of independent metabolites is not given simply by rank(*N*), as is generally (and erroneously) suggested; rather the local dynamics of the system must also be taken into account via the elasticity matrix. The conservations may be removed through matrix decomposition, using a *m × m*_0 _link matrix *L *that relates the complete vector of internal metabolites to the vector of independent metabolites [28]. Writing *A *= *N ε*' and letting *A*_*r *_denote a *m*_0 _× *m *matrix composed of linearly independent rows of *A*, the corresponding link matrix is defined as , where '^+^' denotes the Moore-Penrose pseudoinverse [29]; hence *A = L·A*_*r*_.

From Equation (6), and noting that the rows of *L *corresponding to the independent metabolites *x*_*r *_form the identity matrix, we find *x *= *L x*_*r *_and hence(7)

where the *m*_0 _× *m*_0 _matrix (*N*_r_·*ε*'. *L*) is invertible through introduction of the link matrix *L*.

Having transformed the system, we add a small perturbation to reaction *j*(8)

where *δ *is our perturbation; *e*_*j *_denotes the *j*^th ^standard basis vector and the notation *N*_*r*, *j *_is used to denote the *j*^th ^column of *N*_*r*_. The new steady state resulting from this perturbation is given by(9)

Using Equation (9), we may resolve the definition of (unscaled) flux control and concentration control coefficients as(10)

and(11)

respectively. If we compare our expressions to those given in Reder [22], we see that they are identical, save in her case *r' *is defined as the independent rows of *N*, leading to . If *r *= *r' *(*i.e*. if rank(*N ε'*) = rank(*N*)), then *L *= *L' *and the two results are equivalent.

As such, we may see that we have extended Reder's work to encompass the possibility that rank(*N ε'*) < rank(*N*), as is the case for our model (rank(*N ε'*) = 205, whilst rank(*N*) = 616). From Equations (10) and (11), one may trivially deduce the summation and connectivity theorems.

Equation (10) may be used to calculate flux control coefficients for our genome-scale model. These parameters may also be defined in their more usual scaled form(12)

### Nomenclature

The indices and variables appearing throughout the paper are defined in Table 6.

**Table 6:** Nomenclature

Index	Description	Size
*i*	species/metabolites	*m*
*j*	reactions	*n*
*BM*	subset of *j*: reactions with fluxes defined in BioModels	55
*r*	subset of *i*: all independent metabolites	*m*_0_

**Variable**	**Description**	**Dimensions**

*A*	*N*·*ε'*	*m × m*
*c*	compartment volumes	*m × 1*
*C*^*J*^	scaled flux control coefficients	*n × n*
*C*^*J*^*'*	unscaled flux control coefficients	*n × n*
*C*^*S*^*'*	unscaled concentration control coefficients	*m × n*
*e*_*j*_	denotes the *j*^th ^standard basis vector	*n × 1*
*f*	vector specifying the optimized fluxes	*n × 1*
*N*	stoichiometric matrix	*m × n*
*L*	link matrix	*m × m*_0_
*t*	time	
*x*	metabolite concentrations	*m × *1
x*	reference metabolite concentrations	*m × *1
*x*_*r*_	independent metabolite concentrations	*m*_0 _× 1
*v*	flux vector	*n × *1
v*	reference flux vector	*n × *1
*v*^min^	lower bounds vector	*n × *1
*v*^max^	upper bounds vector	*n × *1
*v*^*T*^	fluxes defined in the Biomodels database	55 × 1
*Z*	optimization objective	
Z*	maximum achievable growth rate	
*Z*_1_	minimal total flux through the network	
*δ*	perturbation	
*ε*	elasticity	*m × n*
*ε*'	unscaled elasticity matrix	*m × n*

## Authors' contributions

KS performed the calculations. KS and ES drafted the manuscript. All authors conceived the methodology and read and approved the final manuscript.

## Supplementary Material

Additional file 1 **Genome-scale model for yeast**. Compressed ZIP file (220 KB) containing the model in SBML format. Click here for file

Additional file 2 **Reference fluxes**. Excel spreadsheet (XLS, 105 KB) containing the reference flux for all reactions, as estimated by application of the algorithm [14]. Click here for file

Additional file 3 **Control over glucose transport**. Excel spreadsheet (XLS, 105 KB) containing flux control coefficients for all reactions for control over glucose transport. Click here for file

Additional file 4 **Control over biomass production**. Excel spreadsheet (XLS, 114 KB) containing flux control coefficients for all reactions for control over biomass production. Click here for file
